# Asymptomatic, Incidental Quadricuspid Aortic Valve: A Case Report and Discussion of Management

**DOI:** 10.7759/cureus.59454

**Published:** 2024-05-01

**Authors:** Nicole Mamprejew, Alex Ashkin, Aleatha Reitsma, David Axline

**Affiliations:** 1 Graduate Medical Education/Internal Medicine, Naples Comprehensive Health (NCH) Healthcare System, Naples, USA; 2 Cardiology, Naples Comprehensive Health (NCH) Rooney Heart Institute, Naples Comprehensive Health (NCH) Healthcare System, Naples, USA

**Keywords:** echocardiography, cardiovascular imaging, aortic valve repair, aortic valve replacement, aortic insufficiency, aortic regurgitation, valvular heart disease, quadricuspid aortic valve

## Abstract

With its exceeding rarity, there is little research on the quadricuspid aortic valve (QAV) inherently to drive guideline-based management. This leaves physicians without evidence-based guidance on the management of such patients should they come across this finding on imaging or should they care for a symptomatic patient. This article describes the case of an incidentally identified QAV in a patient undergoing treatment for tuberculosis, which seemingly had bicuspid-appearing valve hemodynamics. Additionally, current literature is reviewed to describe classification, presentation, complications, and intervention, with additional exploration and commentary on the lack of guideline-based care.

## Introduction

A quadricuspid aortic valve (QAV) is a four-leaflet congenital variant of the aortic valve. QAVs are exceedingly rare, with incidence around 0.006% to 0.043% [1-3]. The aortic valve originates from mesenchymal ridges of the truncus arteriosus during the fifth to ninth weeks of embryogenesis [1,4]. It is postulated that a QAV may arise during various points of embryogenesis, including division of the conotruncus, valve cushions, or mesenchymal ridge, but the particular etiology remains to be elucidated [1-2,4]. The QAV has been characterized by two classification systems, with the most widely cited being the Hurwitz and Roberts system [2-3]. In this system, there are seven subtypes classified by the cusp size; subtypes A through C encompass 85% of phenotypes [3,5]. The Nakamura classification system characterizes the QAV into four variants based on relative positioning of the supernumerary leaflet [6].

Presentation ranges from asymptomatic with an incidental finding of QAV on imaging, up to a spectrum of heart failure symptoms [5]. Several clinical complications are associated with a QAV. The most common valvular derangement is aortic regurgitation (AR) [1]. The proposed mechanism of AR is unequal shear stress on the leaflets, leading to their progressive fibrosis eventually resulting in poor leaflet coaptation [2,7]. A study by the Mayo Clinic which reviewed echocardiographic data of fifty patients with QAVs found moderate to severe AR in 26% of participants [1]. Aortic stenosis was less prevalent, found only in 8% of participants [1]. Mild aortic dilatation was noted in 79% of the study population, though patients experienced little to no significant progression of dilatation during the follow-up period, with no dissection or rupture documented [1]. Additionally, a feared complication of valvular dysfunction is infective endocarditis (IE). Despite the increased propensity for IE in the bicuspid aortic valve (BAV) [8], only a few cases of IE are described in patients with a QAV [1]. In the Mayo Clinic study, the incidence of IE was zero [1]. This raises a significant divergence from the BAV, which increases the risk of IE 8-fold [1,9]. The American College of Cardiology/American Heart Association (AHA/ACC) 2020 IE guidelines have no recommendations for antibiotic prophylaxis in QAVs, nor is a QAV a qualifying indication [10].

This article presents a case of an incidentally discovered, asymptomatic QAV in a patient with tuberculosis and additionally discusses the gap of guideline-based care in the longitudinal management of patients with a QAV. This article was presented as a poster at the National Meeting for the American Federation for Medical Research in Reston, Virginia on October 27, 2023.

## Case presentation

**Figure 1:**
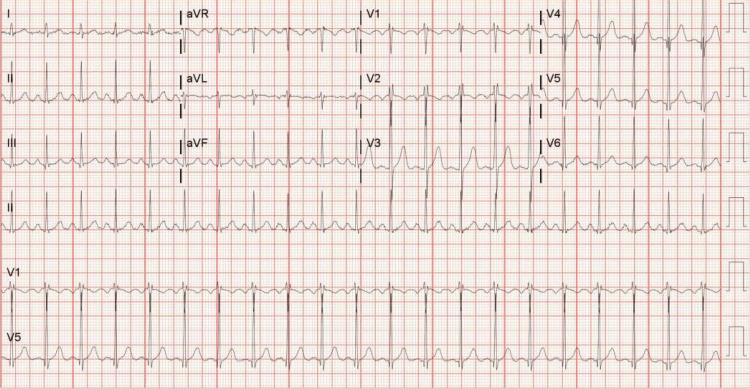
Electrocardiogram The patient’s electrocardiogram on admission, evidencing sinus tachycardia.

**Figure 2:**
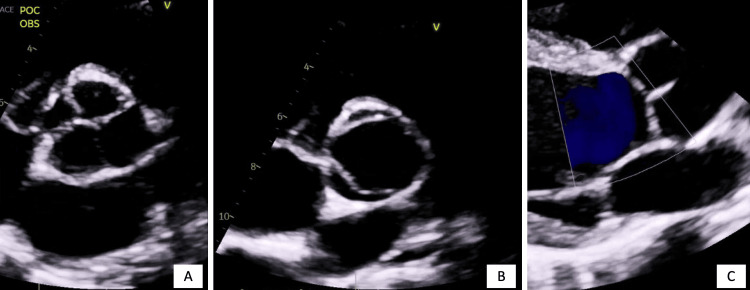
Quadricuspid Aortic Valve Imaging Panel A shows transthoracic echocardiography demonstrating the quadricuspid aortic valve in a closed position with leaflets approximated, in the parasternal short axis view; note the leaflet sizes with one large cusp, two equally sized cusps, and one small cusp, indicative of Hurwitz and Roberts class D. Panel B demonstrates the quadricuspid aortic valve in the open position in the parasternal short axis view. Interestingly, in this view the valve morphology appears bicuspid. Panel C demonstrates the quadricuspid aortic valve parasternal long axis view, demonstrating the lack of aortic regurgitation, evidencing appropriate approximation of the valve leaflets upon closure.

Presented is a 32-year-old male originally from Cuba, with no known past medical history. He presented to the hospital with fever, night sweats, cough, hemoptysis, and 20-25 pounds of weight loss over 2 to 3 months. Acid-fast bacillus smear and culture revealed *Mycobacterium tuberculosis*. He was initiated on rifamycin, isoniazid, pyrazinamide, and ethambutol (RIPE). Incidentally, during his hospital stay, it was revealed that as a child in Cuba, the patient was told of an underlying heart condition but was unaware of a formal diagnosis; he had no further workup at the time. The patient denied chest pain, lower extremity swelling, orthopnea, or palpitations. Vital signs on admission revealed a temperature of 37.7 degrees Celsius, a heart rate of 135 beats per minute (Figure 1), a respiratory rate of 18 respirations per minute, a blood pressure of 142/99 mmHg, and saturating 99% on room air. On physical exam, he was tachycardic with a regular rhythm, no murmur, no jugular venous distention, and no lower extremity edema. On initial labs, his electrolytes, kidney function, and hepatic function were within normal limits, but leukocytosis was present with a white blood cell count of 15,100 per microliter. Serial high-sensitivity troponins were negative, and an electrocardiogram revealed sinus tachycardia with evidence of left atrial and left ventricular (LV) enlargement. A transthoracic echocardiogram (TTE) revealed a Hurwitz and Roberts class D quadricuspid aortic valve with no evidence of AR (Figure 2). LV ejection fraction was 40-45% with moderately decreased global LV systolic function. LV fractional shortening was decreased at 18%. The aortic root and ascending aorta were without dilatation. The right and left atria, as well as the right ventricle, were normal. The mitral and pulmonic valves had normal anatomy and function; however, the tricuspid valve had trace regurgitation. Pulmonary artery systolic pressure was normal with an estimated right ventricular systolic pressure of 21 mmHg. 

A transesophageal echocardiogram was deferred given the patient’s infection with no evidence of acute heart failure to warrant further inpatient evaluation. Fortunately, he continued to show improvement in his underlying pulmonary symptoms and was discharged from the hospital on RIPE therapy with outpatient cardiology follow-up recommended.

## Discussion

The presented patient’s aortic valve anatomy was consistent with a Hurwitz and Roberts class D phenotype, without any AR or aortic dilatation. Interestingly, the patient’s valvular anatomy appeared bicuspid in the opened position, which begs the question of whether QAV classification affects the dynamics of the leaflets, which seems yet to be studied. Consequently, with some QAVs seemingly behaving like a BAV, this also begs the question of whether guidelines for management should mirror that of BAVs in special cases. Further research is needed into valve hemodynamics in this rare anomaly, especially since there is little consensus on the management of QAVs given their rarity. This poses physicians with a challenge regarding the longitudinal care of patients with QAVs. A reasonable proposal would be that management should mirror AR guidelines from the 2020 AHA/ACC Guideline for the Management of Patients With Valvular Heart Disease, given that it is the most common valvular complication. 

Mirroring the AR guidelines, in patients with a QAV but no evidence of insufficiency, they necessitate an annual history and physical exam with repeat imaging should they have an onset of symptoms or change in exam [10]. A TTE every 3-5 years for continued surveillance is also necessitated [10]. In the case of the presented patient, appropriate follow-up of the QAV with TTE would be recommended in 3-5 years, given his absence of symptoms and absence of AR. However, his heart failure with mildly reduced ejection fraction at 40-45% would require reevaluation for improvement or worsening of the ejection fraction, with further discussion beyond the scope of this article. Otherwise, in patients with AR who have mild severity, TTE is recommended every 3-5 years, in moderate severity every 1-2 years, and in severe cases every 6-12 months [10]. 

Patients may be offered aortic valve replacement or repair, and those with QAVs who require surgical intervention are interestingly diagnosed with a QAV nearly a decade earlier than those who never require surgical intervention [1,5,11]. In the Mayo Clinic study, only 16% of the patient population required aortic valve surgery [1]. Following the AHA/ACC guidelines for AR, there is a class I indication for valve intervention in severe, symptomatic AR, irrespective of systolic function [10]. Also, in patients who are asymptomatic but have severe AR and LV ejection fraction below 55%, there is a class I indication for valve intervention if there is no other attributable factor for systolic dysfunction [10]. The average age at which intervention takes place is the fifth or sixth decade of life [1,12]. Patients with a QAV who require valve intervention are fairly young, highlighting the importance of selecting repair versus replacement [1]. In younger surgical candidates, QAV repair is preferred to replacement to avoid known complications, most commonly being prosthetic valve degeneration, endocarditis, thromboembolism, and bleeding [13]. 

In terms of survival, across studies, the five-year survival has been noted around 89.9% to 91.5%, and the 10-year survival rate is 84.9% to 87.7% [1-2]. Those who undergo surgery also have favorable long-term outcomes whether they undergo aortic valve repair or replacement [1]. The Mayo Clinic study notes no significant differences in survival between the patients who underwent valve surgery compared to those who did not require surgical intervention [1]. This portends a good prognosis for the presented patient. 

## Conclusions

In conclusion, this case report aimed to inform physicians about the rare QAV, as explored through the presented asymptomatic patient with an incidentally identified QAV. The low prevalence of the QAV poses limitations in management due to a lack of guidelines. Further research is needed to fully describe the congenital variant, especially as it seems the valve may have varying hemodynamics based on classification; however, it can be argued that it is reasonable to follow the guidelines for AR for surveillance and treatment given it is the most common valvular complication. 
